# Interventions to improve quality of life for informal caregivers of people with dementia nearing the end of life: A meta‐review and system‐based logic model

**DOI:** 10.1002/alz70858_101517

**Published:** 2025-12-25

**Authors:** Alex Hughes, Yuxin Zhou, Kirsten Moore, Tofunmi Aworinde, Nathan Davies, Clare Ellis‐Smith, Katherine Sleeman, Elizabeth Lesley Sampson, Charlotte Kenten, Yolanda Barrado‐Martin, Catherine Henderson, Victoria Vickerstaff, Catherine Evans

**Affiliations:** ^1^ King's College London, London, United Kingdom; ^2^ University College London, London, United Kingdom; ^3^ Queen Mary University of London, London, London, United Kingdom; ^4^ London School of Economics and Political Science, London, United Kingdom

## Abstract

**Background:**

Informal caregivers play a critical role in supporting people with dementia nearing the end of life and are at risk of stress, burden and depression. Despite this, evidence gaps exist on the optimal interventions for caregivers. This meta‐review aimed to identify, appraise and synthesise evidence on interventions designed to improve the quality of life of informal caregivers.

**Method:**

Epistemonikos, MEDLINE, and ASSIA were searched for reviews published between 1980 and April 2024. Quality was assessed using AMSTAR 2, and a narrative synthesis was performed. Analysis was conducted through a palliative care lens and involved charting concepts and components of dementia palliative care to caregiver interventions. Outcomes were then categorised based on whether dementia care or palliative care was the main approach and then linked to intervention concepts and evidence strength. Implementation requirements were mapped to the Consolidated Framework for Implementation Research (CFIR), culminating in the development of a system‐based logic model.

**Result:**

Ten systematic reviews, covering 138 primary studies with 4,000 participants were included. Seven reviews had palliative care as the main approach and three were dementia focused. Interventions were grouped into three categories: psychosocial, educational and decision‐making. These interventions aligned with almost all the identified key concepts of dementia palliative care, particularly holistic assessments, person‐centered care and education. Caregiver outcomes were identified and grouped into five categories: (1) psychosocial, (2) skills (3) relationships, (4) communication, and (5) care planning. Palliative care focused reviews emphasised communication and decision‐making, whereas dementia focused reviews were more concerned with skills and coping. Meta‐analyses supported educational interventions in reducing depression, and decision‐making aids in lowering decisional conflict. The overall evidence quality was low and the reported strategies for implementing interventions were limited. A logic model was developed which detailed the essential context, processes and outcomes for implementing integrated dementia palliative care.

**Conclusion:**

Integrated dementia palliative care for caregivers requires multifaceted interventions. Combining dementia care and palliative care approaches addresses the distinct yet complementary concepts of dementia palliative care, while also impacting a broader range of caregiver outcomes and offering a more holistic approach. Future research is required on developing standardised outcome measures, cost effectiveness and implementation.

